# Is Passynski’s Approach to Hydration Numbers Consistent with Thermodynamics?

**DOI:** 10.3390/molecules29174214

**Published:** 2024-09-05

**Authors:** Wojciech Marczak

**Affiliations:** Faculty of Science and Technology, Jan Dlugosz University, Al. Armii Krajowej 13/15, 42-200 Częstochowa, Poland; w.marczak@ujd.edu.pl

**Keywords:** solvation, hydration, aqueous solutions, compressibility, Pasynski, speed of sound, ideal mixture

## Abstract

Hydrophilic and hydrophobic phenomena occur in aqueous solutions. Despite the complex nature of the molecular interactions, the propensity of molecules and ions to hydration is sometimes characterized by a single “hydration number”. Passynski’s method for determining the hydration numbers in dilute aqueous solutions belongs to the group of methods based on the analysis of the isentropic compressibility of a mixture. Isentropic compressibility is a thermodynamic material constant; thus, the paper deals with Passynski’s approach discussed in terms of thermodynamics. First, Passynski’s assumptions were applied to the volume of the mixture. Subsequent strict thermodynamic derivation led to a formula for the hydration number which resembled that of Onori rather than the original one. Passynski’s number turned out to be inconsistent with the thermodynamics and mechanics of fluids. This is a rather purely empirical measure of the slope of the dependence of isentropic compressibility on the solute mole fraction in a dilute aqueous solution. Being the quotient of the slope and the isentropic compressibility of pure water, Pasynski’s numbers are more convenient to analyze and discuss than the slopes themselves. Conclusions about molecular interactions based on these numbers must be treated with considerable caution.

## 1. Introduction

The lack of an unequivocal method for determining the molecular order in liquids causes many tentative concepts to exist even if they are not clearly defined. One such is that of the solvation number. Contrary to crystals, molecular aggregates in the liquid phase are often dynamic, labile, and of variable size. It seems rather difficult, if possible at all, to characterize complex solute-solvent interaction in liquids by just one number. The interactions in aqueous solutions involve hydrogen bonds that are manifested in hydrophilic and hydrophobic phenomena [1]. No wonder the hydration numbers depend not only on the system itself but also on the method of determination. A group of such methods is based on the analysis of the compressibility of aqueous solutions. One of these was suggested by Passynski (also spelled Pasynski) almost a century ago [2,3]. It is still applied (e.g., [3,4,5,6,7,8,9,10,11,12,13,14,15]), although its consistency with the thermodynamics and mechanics of fluids has never been verified. This paper is aimed at filling this gap. 

Various solutes dissolved in water, both electrolytes and non-electrolytes, lower the compressibility of the liquid [3]. Passynski [2,3] assumed that the isentropic compressibility of binary aqueous solution results solely from the compressibility of bulk water, *κ_S_*_,1_^*^ > 0, while both the solute and water in the hydration sphere are incompressible: *κ_S_*_,2_^*^ = 0 and *κ_S_*_,1_^h^ = 0, respectively. The bulk water in the mixture is identical to pure water. Thus, the amount of compressible water decreases with the addition of the solute due to hydration, and each solute molecule binds several water molecules to form the hydrate. This led to the conclusion that the isentropic compressibility of such a mixture relates to that of pure water in the following manner:

(1) $$\kappa_{S}=\kappa_{S,1}^{*}\frac{n_{1}-k_{P}n_{2}}{n_{1}}=\kappa_{S,1}^{*}\frac{x_{1}-k_{P}x_{2}}{x_{1}}$$

where *n_i_* is the number of moles, *x_i_* is the mole fraction, *i* = 1 for water, *i* = 2 for the solute, and *k*_P_ is the hydration number. The latter will be called “Passynski’s number” from here on to avoid confusion with other similar quantities reported in this work. In other words, Passynski arbitrarily assumed that the isentropic compressibility of the system is directly proportional to the quantity of bulk water. It seems questionable at the least. The following re-arranged Equation (1) gives the Passynski’s number:

(2) $$k_{P}=\frac{n_{1}}{n_{2}}\left(1-\frac{\kappa_{S}}{\kappa_{S,1}^{*}}\right)$$

where the molar ratio is equal to the mole fractions ratio: $n_{1}/n_{2}=x_{1}/x_{2}$. Indeed, the *k*_P_ depends on the concentration. For this reason, it is sometimes reported for infinitely diluted solutions [3], calculated as follows:

(3) $$k_{P}^{\infty }=\underset{x_{2}\to 0}{\lim}k_{P}$$

Isentropic compressibility can be calculated from the experimental speed of sound in the liquid, *u*, and its density, *ρ*, using Laplace’s formula as follows:

(4) $$\kappa_{S}=\rho^{-1}u^{-2}$$

Precise and easy-to-operate speed of sound and density meters, e.g., [16], developed in the last decades, facilitated the routine measurements of the speed and density. That tempts researchers to calculate and report Passynski’s numbers identified as hydration numbers [3,4,5,6,7,8,9,10,11,12,13,14,15]. The author believes that this community will appreciate the present evaluation of Passynski’s method.

## 2. Two-State Model of the Binary Aqueous Solution

Equations (1) and (2) seem to result from the formation of incompressible hydrates in the solution. However, different conclusions can be drawn if one considers the volume of the mixture first, and the compressibility only after that. Application of Passynski’s idea of hydration leads to the following formula for the extensive volume of the mixture:

(5) $$V=n_{1}^{*}V_{1}^{*}+n_{2}V_{2}^{h}$$

where *V*_1_^*^ is the molar volume of pure water, *V*_2_^h^ is the molar volume of the hydrate, *n*_1_^*^ is the quantity of bulk water (i.e., number of moles), and *n*_2_ is the quantity of the solute which is equal to the quantity of the hydrate expressed in moles. The molar volume of the hydrate, *V*_2_^h^, encompasses the molar volumes of the solvent and the hydration sphere. The *n*_1_^*^ is related to the total number of water molecules, *n*_1_, and the hydration number, *k*, by the following Equation (6):

(6) $$n_{1}^{*}=n_{1}-kn_{2}$$

Consequently:

(7) $$V=n_{1}V_{1}^{*}-n_{2}kV_{1}^{*}+n_{2}V_{2}^{h}$$

Equation (7) is the fundamental relationship for the volume of Passynski’s mixture. Thus, Passynski’s idea applied to the volume of the mixture led to the equation which is identical to that suggested by Onori [3,17]. 

The pressure derivative of *V* defines the compression of the system as follows:

(8) $$K_{z}\equiv -{\left(\frac{\partial V}{\partial p}\right)}_{z}$$

where *z* is the temperature (*T*) or entropy (*S*) for the isothermal and isentropic processes, respectively. Like *V*, *K_z_* is an extensive property. The compressibility is its intensive counterpart:

(9) $$\kappa_{z}=\frac{K_{z}}{V}=-\frac{1}{V}{\left(\frac{\partial V}{\partial p}\right)}_{z}$$

Isentropic compressibility has been used in the calculations of Passynski’s numbers. Now, we shall prove that Passynski’s Equation (2) cannot be obtained from the model Equation (7). 

Substitution of Equation (7) to Equation (8) gives:

(10) $$K_{z}=-n_{1}{\left(\frac{\partial V_{1}^{*}}{\partial p}\right)}_{z}+n_{2}k{\left(\frac{\partial V_{1}^{*}}{\partial p}\right)}_{z}+n_{2}V_{1}^{*}{\left(\frac{\partial k}{\partial p}\right)}_{z}-n_{2}{\left(\frac{\partial V_{2}^{h}}{\partial p}\right)}_{z}$$

which for isothermal conditions reduces to the following:

(11) $$K_{T}=n_{1}K_{T,1}^{*}-n_{2}kK_{T,1}^{*}+n_{2}V_{1}^{*}{\left(\frac{\partial k}{\partial p}\right)}_{T}-n_{2}{\left(\frac{\partial V_{2}^{h}}{\partial p}\right)}_{T}\;$$

Following Passynski, we assume that the hydrate is incompressible, ${\left(\partial V_{2}^{h}/\partial p\right)}_{T}=0$, and the hydration number *k* does not depend on pressure, *k* = const. The latter is counterintuitive. One would expect that an increase in pressure should result in a disruption of the lattice-like structure of the bulk water and the growth of the incompressible hydration sphere. However taking this for granted, one obtains the following:

(12) $$K_{T}=n_{1}K_{T,1}^{*}-n_{2}kK_{T,1}^{*}.$$

For 1 mole of the mixture, Equation (12) takes the form:

(13) $$K_{T,m}=x_{1}K_{T,1}^{*}-x_{2}kK_{T,1}^{*}$$

where $K_{T,m}=K_{T}/\left(n_{1}+n_{2}\right).$ Re-arranging Equation (13) gives the hydration number *k*:

(14) $$k=\frac{1-K_{T,m}/K_{T,1}^{*}}{x_{2}}-1$$

or

(15) $$k=\frac{1-\left(V_{m}\kappa_{T}\right)/\left(V_{1}^{*}\kappa_{T,1}^{*}\right)}{x_{2}}-1$$

where *V*_m_ is the molar volume of the mixture, *κ_T_* is its isothermal compressibility, and *V*_1_^*^ and *κ_T_*_,1_^*^ are the respective functions for pure water.

Now, a question arises whether Equations (14) and (15) are valid for *Κ_S_* and *κ_S_* substituted for their isothermal counterparts. Equation (10) for the isentropic conditions and *k* = const. would take a form similar to the respective equation in Onori’s model [3,17], in which ${\left(\frac{\partial V_{2}^{h}}{\partial p}\right)}_{S}\neq 0$. Of crucial importance here is noticing the fundamental difference between isothermal and isentropic compressions, *K_T_* and *K_S_*. Only the first one is a Gibbsian property [18], which makes a substitution of *K_T_* for $-{\left(\partial V/\partial p\right)}_{T}$ always correct. Similar substitution of *K_S_* for $-{\left(\partial V/\partial p\right)}_{S}$ is not possible for the derivatives of volume on the right-hand side of Equation (10). For example:

(16) $$K_{S,1}^{*}=-{\left(\frac{\partial V_{1}^{*}}{\partial p}\right)}_{S_{1}^{*}}$$

rather than just

(17) $$K_{S,1}^{*}=-{\left(\frac{\partial V_{1}^{*}}{\partial p}\right)}_{S}.$$

Indeed, the quantities given by Equations (16) and (17) differ one from another. Moreover, Equation (17) is ambiguous. Subscript *S*_1_^*^ in Equation (16) denotes the constant partial entropy of the first component of the system, i.e., the bulk water here. The conditions in Equation (17) are less restrictive—subscript *S* indicates that just the total entropy of the system remained unchanged:

(18) $$S={n_{1}^{*}S}_{1}^{*}+n_{2}S_{1}^{h}$$

This does not imply *S*_1_^*^ = const. for *S* = const. On the contrary, the changes in entropy of the mixture components, *n*_1_*S*_1_^*^ and *n*_2_*S*_1_^h^, may cancel each other out resulting in the constant total entropy *S*. For the same reason the molar isothermal compression of the thermodynamic ideal mixture, *K_T_*_,m_^id^, is the mole-fraction weighted average of the isothermal compressions of its pure components, calculated as follows:

(19) $$K_{T,m}^{id}=\sum_{i=1}^{\alpha }x_{i}K_{T,i}^{*}$$

while for the isentropic compression:

(20) $$K_{S,m}^{id}\neq \sum_{i=1}^{\alpha }x_{i}K_{S,i}^{*}$$

A well-known thermodynamic relationship relates the two compressions one to another, e.g., [19]:

(21) $$K_{T,m}=K_{S,m}+T\frac{E_{m}^{2}}{C_{p,m}}$$

where $E_{m}={\left(\partial V_{m}/\partial T\right)}_{p}$ is the isobaric molar thermal expansion and *C_p_*_,m_ is the isobaric heat capacity. Thus, the substitution of *K_T_*_,m_ and *K_T_*_,1_^*^ given by Equation (21) to Equation (14) relates the hydration number to the isentropic compression. An alternative is the following approximate relationship:

(22) $$k\approx \frac{1-K_{S,m}/K_{S,1}^{*}}{x_{2}}-1=\frac{1-\left(V_{m}\kappa_{S}\right)/\left(V_{1}^{*}\kappa_{S,1}^{*}\right)}{x_{2}}-1$$

which results from the rather bold assumption:

(23) $$T\frac{E_{m}^{2}}{C_{p,m}}=0$$

Equations (14), (15) and (22) differ from Passynski’s Equation (2). 

## 3. Discussion

We have shown that Passynski’s assumption applied to the volume leads to equations different from those suggested by himself. The two fundamental reservations about Passynski’s reasoning are (*i*) consideration of the isentropic rather than isothermal compressibility and (*ii*) the additivity rules for compressibility inconsistent with thermodynamics and mechanics of fluids. Indeed, the derived model of the mixture compressibility is not Passynski’s Equation (1) but that of Onori [17], simplified by the assumption about the incompressible hydration sphere. Passynski’s numbers (Equation (2)) are arbitrary rather than resulting from a thermodynamically justified model because the isentropic compressibility of the binary ideal mixture of the incompressible and compressible species is not the mole-fraction-weighted average of the two quantities [19]. 

A good illustration could be the compressibility of the thermodynamic ideal binary mixture of a monoatomic gas with a diatomic homonuclear one. Both pure gases and their mixture fulfill the equation of state:

(24) $$pV_{m}=RT$$

where *R* is the universal gas constant. The isothermal compressibility of the two gases, as well as their mixtures, is *κ_T_* = *p*^−1^. The pure gases differ only in the isobaric heat capacities, equal to 5/2*R* and 7/2*R*, respectively. The isentropic compressibility can be calculated from the modified Equation (21), written as follows:

(25) $$\kappa_{S}=\kappa_{T}-T\frac{V_{m}\alpha^{2}}{C_{p,m}}$$

where $\alpha =T^{-1}$, *V*_m_ = *V*_1_^*^ = *V*_2_^*^, and *C_p_*_,m_ follow the ideal mixing rule:

(26) $$C_{p,m}=x_{1}C_{p,1}^{*}+x_{2}C_{p,2}^{*}$$

**Figure 1:**
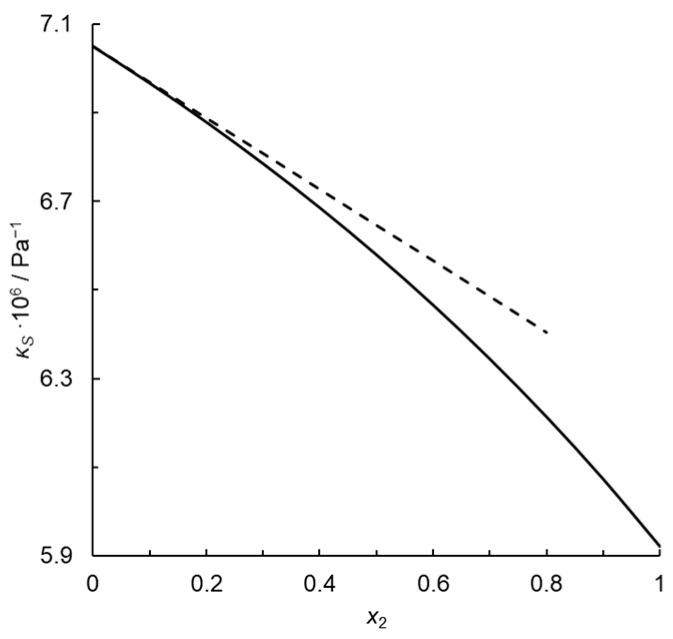
Isentropic compressibility of the thermodynamic ideal binary mixture of a monoatomic gas with a diatomic homonuclear one at *T* = 273.15 K and *p* = 101,325 Pa (solid line), and tangent to this curve at *x*_2_ = 0 (dashed line); *x*_2_—mole fraction of the monoatomic gas.

Passynski’s number calculated for the monoatomic gas infinitely diluted in the diatomic one is 0.114 rather than 0, which would be expected for a fully random distribution of molecules. Moreover, Passynski’s number depends on concentration because isentropic compressibility does not depend rectilinearly on the mole fraction, as illustrated in Figure 1. The equation analogous to Passynski’s Equation (2) but with isothermal compressibility instead of an isentropic one is as follows:

(27) $$k_{P}^{\prime }=\frac{n_{1}}{n_{2}}\left(1-\frac{\kappa_{T}}{\kappa_{T,1}^{*}}\right)$$

where *k*_P_^′^ = 0, because *κ_T_* = const. in the whole concentration range. 

**Figure 2:**
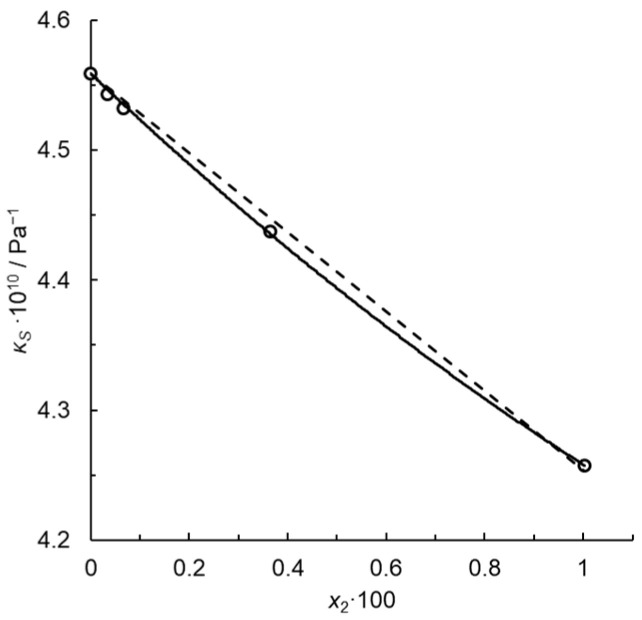
Isentropic compressibility of the system water (1) + *N*-methylpiperidine at *T* = 293.15 K. Points—calculated using Equation (4) from the speeds of ultrasound and densities reported in [20]; solid line—Equation (31); dashed line—Equation (28).

The above considerations suggest that Passynski’s number is not a sensical characteristic of hydration. On the other hand, Passynski’s approach gave intuitively acceptable results for homologous series of alcohols, polyols, carbohydrates, polymers, and amino acids [3]. Burakowski and Gliński [3] thoroughly discussed this and five other methods of calculating the “hydration numbers” from the isentropic compressibility of dilute aqueous solutions or directly from the speed of sound in such systems. They noted that the isentropic compressibility of dilute solutions of nonelectrolytes (*x*_2_ < 0.01) can be satisfactorily interpolated by an empirical equation:

(28) $$\kappa_{S}=ax_{2}+\kappa_{S,1}^{*}$$

where *κ_S_*_,1_^*^ is a constant parameter, the coefficient *a* can be obtained by the least squares regression, and $a=\frac{d\kappa_{S}}{dx_{2}}$. The *κ_S_* expressed by Equation (28) substituted to Equation (2) gives the Passynski’s number, calculated as follows:

(29) $$k_{P}=-x_{1}\frac{d\kappa_{S}/dx_{2}}{\kappa_{S,1}^{*}}$$

for *x*_2_ < 0.01, and

(30) $$k_{P}^{\infty }=\underset{x_{1}\to 0}{\lim}k_{P}=-\frac{{\left(d\kappa_{S}/dx_{2}\right)}_{x_{2}=0}}{\kappa_{s,1}^{*}}$$

for the infinitely dilute solution. Note that *k*_P_ for *x*_2_ = 0.01, i.e., for the maximum solute concentration, is just 1% lower than $k_{P}^{\infty }$, because $d\kappa_{S}/dx_{2}=const.$ in this approximation. Thus, *k*_P_ is constant in this concentration range within the uncertainty of the fit limits. Figure 1 illustrates the fit for dilute solutions of *N*-methylpiperidine in water at a temperature *T* = 293.15 K. Passynski’s number calculated using the linear Equation (28) is 6.69 ± 0.54 at the confidence level of 0.95 for infinite dilution, and determination coefficient *R*^2^ = 0.9961. Despite a high *R*^2^, the dependence of *κ_S_* on *x*_2_ is not rectilinear but rather a convex downward function, approximated by a parabola in Figure 2.

(31) $$\kappa_{S}=bx_{2}^{2}+ax_{2}+\kappa_{S,1}^{*}$$

with constant parameter *κ_S_*_,1_^*^ and fitted coefficients *a* and *b*. Here, no systematic deviations from the regression line occur and the determination coefficient is much higher, *R*^2^ = 0.9995. For infinite dilution, we write the following:

(32) $$k_{P}^{\infty }=-\frac{a}{\kappa_{S,1}^{*}}$$

and $k_{P}^{\infty }$ = 7.88 ± 1.39 at the confidence level of 0.95. Such convex functions are typical of the isentropic compressibility isotherms for dilute aqueous solutions, e.g., [15]. Thus, Passynski’s numbers depend on the function applied to interpolate the empirical dependency of *κ_S_* on *x*_2_. Their values must be calculated using carefully selected interpolation functions which provide random distribution of the residual deviations. 

Equations (29) and (30) illustrate the fact that Passynski’s number is just an empirical characteristic of the slope of the *κ_S_*(*x*_2_) function. However, Passynski’s number seems to be related to the molecular structure of the solute. For example, it increases by 1.0 per methylene group in normal alcohol molecules, while by 0.75 in those of α,ω-diols [3]. Noteworthy is that other “hydration numbers” defined by Shiio [21,22,23], Yasunaga [24,25], Isemura and Goto [26,27], Millero [28,29], and Onori [17], and discussed by Burakowski and Gliński [3], do not fulfill the condition of thermodynamic consistency also. 

## 4. Conclusions

Passynski’s numbers, often called the “hydration numbers” are inconsistent with thermodynamics and the mechanics of fluids. Equation (2) for Passynski’s number does not result from the equation for the volume of the binary mixture based on his model assumptions. The latter resembles rather the equation suggested by Onori [17]. A simple proof for this inconsistency is that Passynski’s number for the solute in the ideal binary mixture of perfect gases differs from zero for the gases with different heat capacities. 

Passynski’s number seems to be a purely empirical measure of the slope of the dependence of isentropic compressibility on the solute mole fraction in dilute aqueous solution (Equations (29) and (30)). The quotient of the slope and the isentropic compressibility of pure water are indeed more convenient to analyze and discuss than the slope itself because it is commonly a small number between one and ten. It is seldom smaller than one, e.g., 0.9 for hydrogen peroxide [3]. Doubtless, hydration affects compressibility and is related to the slope of the *κ_S_*(*x*_2_) function in this way. However, the effects of structure-making and structure-breaking effects of solute molecules in water cannot be explained by such a simple model. Muller’s observation made almost 40 years ago is still worth remembering: “Whether or not a particular solute is perceived as a structure maker thus depends on what data are chosen as a basis for judgment and on personal preferences as to how the data should be construed.” [30].

## Data Availability

Data are included within the article.
